# Differential Lung Protective Capacity of Exosomes Derived from Human Adipose Tissue, Bone Marrow, and Umbilical Cord Mesenchymal Stem Cells in Sepsis-Induced Acute Lung Injury

**DOI:** 10.1155/2022/7837837

**Published:** 2022-02-27

**Authors:** Huimin Deng, Lina Zhu, Yiguo Zhang, Li Zheng, Song Hu, Wenyu Zhou, Tong Zhang, Wenting Xu, Yuanli Chen, Huanping Zhou, Quanfu Li, Juan Wei, Hao Yang, Xin Lv

**Affiliations:** ^1^Department of Anesthesiology, Shanghai Pulmonary Hospital, Tongji University School of Medicine, Shanghai, China; ^2^Wannan Medical College Graduate School, Wuhu, China; ^3^Department of Anesthesiology, Fuyang Hospital of Anhui Medical University, Fuyang, China

## Abstract

Exosomes derived from human mesenchymal stem cells (hMSCs) have the capacity to regulate various biological events associated with sepsis-induced acute respiratory distress syndrome (ARDS), including cellular immunometabolism, the production of proinflammatory cytokines, allowing them to exert therapeutic effects. However, little is known about which type of hMSC-derived exosomes (hMSC-exo) is more effective and suitable for the treatment of sepsis-induced ARDS. The purpose of this study is to compare the efficacy of hMSC-derived exosomes from human adipose tissue (hADMSC-exo), human bone marrow (hBMMSC-exo), and human umbilical cord (hUCMSC-exo) in the treatment of sepsis-induced ARDS. We cocultured lipopolysaccharide- (LPS-) stimulated RAW264.7 macrophage cells with the three kinds of hMSCs and found that all hMSCs reduced the glycolysis level and the content of lactic acid in macrophages. Accordingly, the expression of proinflammatory cytokines also decreased. Notably, the protective effects of hMSCs from adipose tissue were more obvious than those of bone marrow and umbilical cord hMSCs. However, this protective effect was eliminated when an exosome inhibitor, GW4869, was added. Subsequently, we extracted and cocultured hMSC-derived exosomes with LPS-stimulated RAW264.7 cells and found that all three kinds of exosomes exerted a similar protective effect as their parental cells, with exosomes from adipose hMSCs showing the strongest protective effect. Finally, an experimental sepsis model in mice was established, and we found that all three types of hMSCs have obvious lung-protective effects, in reducing lung injury scores, lactic acid, and proinflammatory cytokine levels in the lung tissues and decreasing the total protein content and inflammatory cell count in the bronchoalveolar lavage fluid (BALF), and also can attenuate the systemic inflammatory response and improve the survival rate of mice. Intravenous injection of three types of hMSC-exo, in particular those derived from adipose hADMSCs, also showed lung-protective effects in mice. These findings revealed that exosomes derived from different sources of hMSCs can effectively downregulate sepsis-induced glycolysis and inflammation in macrophages, ameliorate the lung pathological damage, and improve the survival rate of mice with sepsis. It is worth noting that the protective effect of hADMSC-exo is better than that of hBMMSC-exo and hUCMSC-exo.

## 1. Introduction

Sepsis is a common clinical syndrome that can lead to multiple life-threatening organ dysfunctions, including sepsis-induced acute lung injury (ALI), causing high mortality [1, 2]. The concomitant onset of acute respiratory distress syndrome (ARDS), the clinical term for ALI, is a strong risk factor for mortality in patients admitted to the intensive care unit [3]. Currently, the mortality of ARDS patients remains as high as 30-45% due to the scarcity of effective treatments [4]. The pathological process of ARDS is initiated by inflammatory cells, mainly neutrophils and macrophages, that accumulate and infiltrate into the alveolar tissues. This infiltration damages alveolar epithelial cells and capillary endothelial cells, increasing their permeability and leading to diffuse pulmonary interstitial and alveolar edema, which eventually result in refractory hypoxemia and progressive dyspnea [5].

Macrophages can establish an appropriate immune response against invading pathogens in the early stages of sepsis [6]. Macrophages are highly plastic and can show different activation states depending on their immunometabolism, which links the metabolic state of immune cells to their function [7–9]. Several studies from our own group and others have shown that specific metabolic characteristics of macrophages can modulate their polarization state and immune function [9, 10]. Macrophages can be classically activated (M1) when stimulated by lipopolysaccharide (LPS) and *γ*-interferon to produce proinflammatory cytokines such as interleukin- (IL-) 1*β* and tumor necrosis factor- (TNF-) *α*, or they can be alternatively activated (M2) when stimulated by IL-4 to produce anti-inflammatory cytokines such as IL-10 and transforming growth factor [11, 12]. A transition in the polarization state of macrophages from M1 to M2 inhibits the inflammatory response and promotes tissue repair and regeneration [12, 13].

Recent studies have shown that mesenchymal stem cells (MSCs), which are multipotent stem cells derived from a variety of tissues including adipose tissue, bone marrow, and umbilical cord, show promise in the treatment of sepsis-induced ALI [9, 14, 15]. These MSCs can inhibit the M1 polarization of macrophages and promote the switch toward the anti-inflammatory M2 type through regulation of various biological events, including the inhibition of macrophage glycolysis. In a mouse model of LPS-induced sepsis, M2 macrophages exerted anti-inflammatory effects, promoted inflammation resolution and tissue repair, and ultimately alleviated ALI and improved survival [9, 14, 15].

Several studies have confirmed that MSCs perform their biological functions to a large extent by secreting exosomes [17] [18]. Exosomes can deliver lipids, proteins, nucleic acids, and other substances from the original cells to the recipient cells, thereby exerting their biological effects [19]. MSC-derived exosomes have become an area of intense research because of their biosafety and lack of cellular activity that could, for example, decrease the risk of tumorigenesis [19]. MSC-derived exosomes show organ-protective functions that depend on their cellular origin and MSC culture conditions (e.g., seeding density), as well as the method and timing of collection [20]. Although previous work investigated the use of various MSC-derived exosomes in the treatment of sepsis-induced ALI [21–23], cross-sectional comparative studies of the lung-protective capacity of exosomes derived from different human MSCs (hMSCs) in sepsis-induced ALI are lacking. Therefore, the present study is aimed at comparing hMSCs from adipose tissue, bone marrow, or umbilical cord as well as their secreted exosomes for their ability to protect against sepsis-induced ALI in a mouse model.

## 2. Materials and Methods

### 2.1. Animals

Adult male C57BL/6 mice (18-22 g body weight) were purchased from Beijing Vital River Laboratory Animal Technology Co., Ltd. (Beijing, China). All mice were housed in a standard animal care room with a 12 h light/dark cycle and had free access to food and water. All experiments and surgical procedures were approved by the Animal Care and Use Committee of the Tongji University School of Medicine, adhered to the recommendations in the Guide for the Care and Use of Laboratory Animals published by the National Institutes of Health.

### 2.2. Culture and LPS Treatment of RAW264.7 Cells

The macrophage cell line RAW264.7 was purchased from the American Type Culture Collection (Manassas, VA, USA) and grown in Dulbecco's modified Eagle medium (DMEM) (Gibco, CA, USA) supplemented with 10% fetal bovine serum (Gibco) and 1% penicillin/streptomycin (Gibco). RAW264.7 cells were cultured at 37°C with 5% CO_2_. RAW264.7 cells were treated with 1 *μ*g/mL LPS (L3024, Sigma, MO, USA) for 24 h.

### 2.3. Culture of hMSCs from Adipose Tissue, Bone Marrow, and Umbilical Cord

The three types of hMSCs were purchased from Cyagen Biosciences (Guangzhou, China) and grown in DMEM/F12 (Gibco) supplemented with 10% fetal bovine serum (Gibco) and 1% penicillin/streptomycin (Gibco). The cells were seeded in 100 mm cell culture dishes (Corning, NY, USA) and cultured at 37°C with 5% CO_2_ and 90% humidity. The medium was changed every three days. Cell passage was performed after cells reached 90% confluence. After discarding the culture medium, the adherent cells were washed twice with phosphate-buffered saline (PBS) and harvested by incubation with 1 mL of 0.25% trypsin and 1 mM ethylenediaminetetraacetic acid for 2 min at 37°C, followed by incubation with 5 mL of complete medium to neutralize the trypsin. Finally, cells were resuspended at a dilution of 1 : 3 in new medium. hMSCs from the three origins at passages 3-5 were used for all subsequent experiments. The identity of hMSCs was confirmed by flow cytometric detection of typical markers of MSCs using FITC-conjugated monoclonal antibody against CD45 and PE-conjugated monoclonal antibody against CD29, as well as their isotype controls (eBioscience, San Diego, CA, USA).

### 2.4. hMSC Differentiation Assays

We examined the ability of the three types of hMSCs to differentiate into multiple lineages under adipogenic conditions using oil red O staining (Sigma-Aldrich, St. Louis, MO) or under osteogenic conditions using alizarin red staining (Sigma-Aldrich). Cells were inoculated into six-well plates (Corning) at 4 × 10^5^ cells/well and cultured until confluence. Adipogenic differentiation medium (Cyagen Biosciences, Guangzhou, China) was used for adipogenic differentiation analysis according to the manufacturer's instructions. The medium was changed every three days. After induction for three weeks, cells were fixed with PBS containing 10% formaldehyde and stained with oil red O. Osteogenic differentiation medium (Cyagen Biosciences) was used for osteogenic differentiation analysis according to the manufacturer's instructions. The medium was changed every three days. After induction for three weeks, the mineralized osteocytes were visualized with alizarin red staining.

### 2.5. Exosome Isolation and Identification

Exosomes were isolated from the culture medium of the three types of hMSC cultures by ultracentrifugation, as previously described [24]. Briefly, hMSCs, after 3-5 passages, were grown to approximately 80-90% confluence. After discarding the culture medium, the cells were washed with PBS for three times. Then, the medium was replaced by DMEM/F12 supplemented with 10% exosome-depleted FBS (Umibio, China), and then, hMSCs were cultured at 37°C with 5% carbon dioxide for 48 h. The culture medium was centrifuged at 300 × g for 20 min, 2,000 × g for 20 min, then 12,000 × g for 30 min at 4°C to eliminate cells and debris. After centrifugation, the supernatants were filtered through a 0.22 mm filter (Millipore, Billerica, MA, USA) to remove microvesicles. The filtrate was ultracentrifuged at 100,000 × g for 90 min at 4°C to deposit the exosomes using a Beckman ultracentrifuge (Beckman Coulter Optima L-80 XP). The extracted exosomes were identified by nanoparticle-tracking analysis and transmission electron microscopy. Exosome markers CD81 and TSG101 were detected by western blot. The protein content of exosomes was quantified using the Micro Bicinchoninic Acid (BCA) Protein Assay Kit (Thermo Fisher Scientific, USA). The extracted exosomes were then resuspended in 100-200 *μ*L of PBS and stored at -80°C.

### 2.6. Coculture of RAW264.7 Cells with hMSCs

A total of 5 × 10^5^ RAW264.7 cells were seeded into the lower chamber of a six-well transwell coculture system (0.4 *μ*m pore size membrane; Corning) and, after 24 h, treated with 1 *μ*g/mL of LPS or PBS (control) for 1 h. Subsequently, LPS-treated RAW264.7 cells were cocultured with hMSCs in the upper chamber for 24 h. The different coculture conditions were as follows: control medium; 5.0 × 10^5^ hMSCs derived from adipose tissue, bone marrow, or umbilical cord; or the same cells pretreated with GW4869 (10 mM), an inhibitor of exosome release [25].

### 2.7. Coculture of RAW264.7 Cells with hMSC-Derived Exosomes

RAW264.7 cells were seeded into a six-well plate at a density of 5 × 10^5^ cells/well and, after 24 h, treated with either LPS (1 *μ*g/mL) or PBS for 1 h. Subsequently, cells were cocultured for 24 h with 100 *μ*g of hMSC-derived exosomes from the three tissue sources (diluted into 100 *μ*L of PBS) or with PBS alone as control.

### 2.8. Sepsis-Induced ALI Animal Model and Treatment with hMSCs or Exosomes

C57BL/6 mice (18-22 g) were randomly divided into several experimental groups (12 mice per group). The control group was intraperitoneally injected with PBS. The LPS+PBS group was intraperitoneally injected with a single dose of 10 mg/kg LPS and then, 1 h later, given 100 *μ*L PBS intratracheally. The LPS+hMSC mice were intraperitoneally injected with the same dose of LPS and then, 1 h later, given 1 × 10^6^ hMSCs from adipose tissue, bone marrow, or umbilical cord in 100 *μ*L PBS intratracheally. Finally, LPS+exo mice were given the same dose of 10 mg/kg LPS and then, 1 h later, 100 *μ*g of exosomes from the three different tissue sources (diluted in 100 *μ*L PBS) intratracheally. All mice were euthanized at 24 h after LPS injection; then, blood, bronchoalveolar lavage fluid (BALF), and lung tissues were sampled.

In survival experiments, a second cohort of mice received the different treatments as stated above, and their survival was recorded every day. The mice were euthanized on day 7 after survival data had been collected.

### 2.9. RNA Extraction and Reverse Transcription Quantitative Real-Time PCR (RT-qPCR)

Total RNA from RAW264.7 cells was extracted using the RNeasy Mini Kit (Qiagen) or from mouse lung tissues using the Trizol reagent (Invitrogen, Calif). Complementary DNAs (cDNAs) were then synthesized using the Prime Script RT Master Mix (Takara, China) according to the manufacturer's instructions. RT-qPCR was performed on a Light Cycler 480 detection system (Roche, Rotkreuz, Switzerland) by using the iTaq universal SYBR Green Super Mix (Bio-Rad, Hercules, CA, USA). *β*-Actin was employed as the endogenous control. Primers were designed using the Primer 5.0 software (http://www.premierbiosoft.com/primerdesign/) and are listed in Table 1. The mRNA expression levels were calculated using the 2^-∆∆Ct^ method.

### 2.10. Western Blot Analysis

In our study, total protein was extracted from RAW264.7 cells or lung tissues using radioimmunoprecipitation assay lysis buffer (RIPA, Thermo Fisher Scientific, USA) containing a protease inhibitor, a phosphatase inhibitor, and phenylmethanesulfonyl fluoride (all from Beyotime, Shanghai, China). The protein content was determined using the BCA Protein Assay Kit (Thermo Scientific, Rockford, IL, USA). Proteins were separated by 10% sodium dodecyl sulphate–polyacrylamide gel electrophoresis and then transferred onto a polyvinylidene fluoride membrane (Millipore, Billerica, MA, USA). Membranes were blocked with 5% nonfat dry milk in Tris-buffered saline and Tween-20 (Beyotime, Shanghai, China) for 1 h at room temperature and then incubated overnight at 4°C in the presence of primary antibodies (diluted 1 : 1,000; Cell Signaling Technology, Danvers, MA, USA) against the following: pyruvate kinase isoform M2 (PKM2), hexokinase 2 (HK2), lactic acid dehydrogenase A (LDHA), or *β*-actin. Then, membranes were incubated with secondary antibodies (diluted 1 : 1,000; Cell Signaling Technology, Danvers, MA, USA) for 2 h at room temperature. Signals were detected using an enhanced chemiluminescence system (Thermo Scientific), and relative protein expression was finally quantified using the Image Lab software (Bio-Rad, USA).

### 2.11. Enzyme-Linked Immunosorbent Assay (ELISA)

Lung tissue (50 mg) was homogenized, diluted in 500 *μ*L of PBS buffer, and assayed for IL-1*β* and TNF-*α* using commercial ELISAs (Abcam, USA) according to the manufacturer's instructions. The same cytokines were also assayed in serum.

### 2.12. Measurement of Lactic Acid

The levels of lactic acid in culture medium, serum, and BALF were measured using a lactic acid assay kit (Jiancheng, Nanjing, China) according to the manufacturer's protocol.

### 2.13. Lung Histopathology and Lung Injury Score Analysis

Lung tissues were fixed with 4% paraformaldehyde for 24 h. Then, fixed lung tissues were paraffin-embedded following standard methods. Subsequently, the paraffin blocks were cut into 5 *μ*m thick sections and stained with hematoxylin and eosin; the sections were observed under a microscope, and the severity of lung tissue injury was scored based on the following criteria [9]: intra-alveolar congestion, intra-alveolar hemorrhage, infiltration of neutrophils in air spaces or vessel walls, and thickness of the alveolar wall/hyaline membrane. Each item was assigned a score from 0 to 4 on a scale from mild to severe, with higher scores indicating more severe injury. The sum of the scores in each item was considered the lung injury score.

### 2.14. Lung Tissue Wet-to-Dry Weight Ratio

After harvesting tissue from the right lung, the wet weight was recorded. Then, the lung tissue was placed in an oven at 80°C for 24 h and weighed until a constant weight, which was recorded as the dry weight. The ratio of wet weight to dry weight of the lung was calculated.

### 2.15. Total Protein Content and Inflammatory Cell Count in BALF

BALF samples were centrifuged at 800 × g for 5 minutes. Total protein in the supernatant was determined using the BCA Protein Assay Kit. The cells were resuspended with 100 *μ*L PBS, and the total cell count was determined under the microscope. Subsequently, cells were stained with Wright-Giemsa (Solarbio, Beijing, China) according to the manufacturer's protocol. Finally, 200 cells in each slide were counted under a light microscope for macrophage quantification.

### 2.16. Statistical Analysis

Statistical analyses were performed using SPSS 17.0 (IBM, Chicago, IL, USA). Data were presented as mean ± standard deviation from at least three independent experiments. Intergroup differences were assessed for significance using one-way ANOVA with a post hoc Tukey test. Differences associated with *p* < 0.05 were considered statistically significant.

## 3. Results

### 3.1. Characterization of hMSCs and hMSC-Derived Exosomes

**Figure 1:**
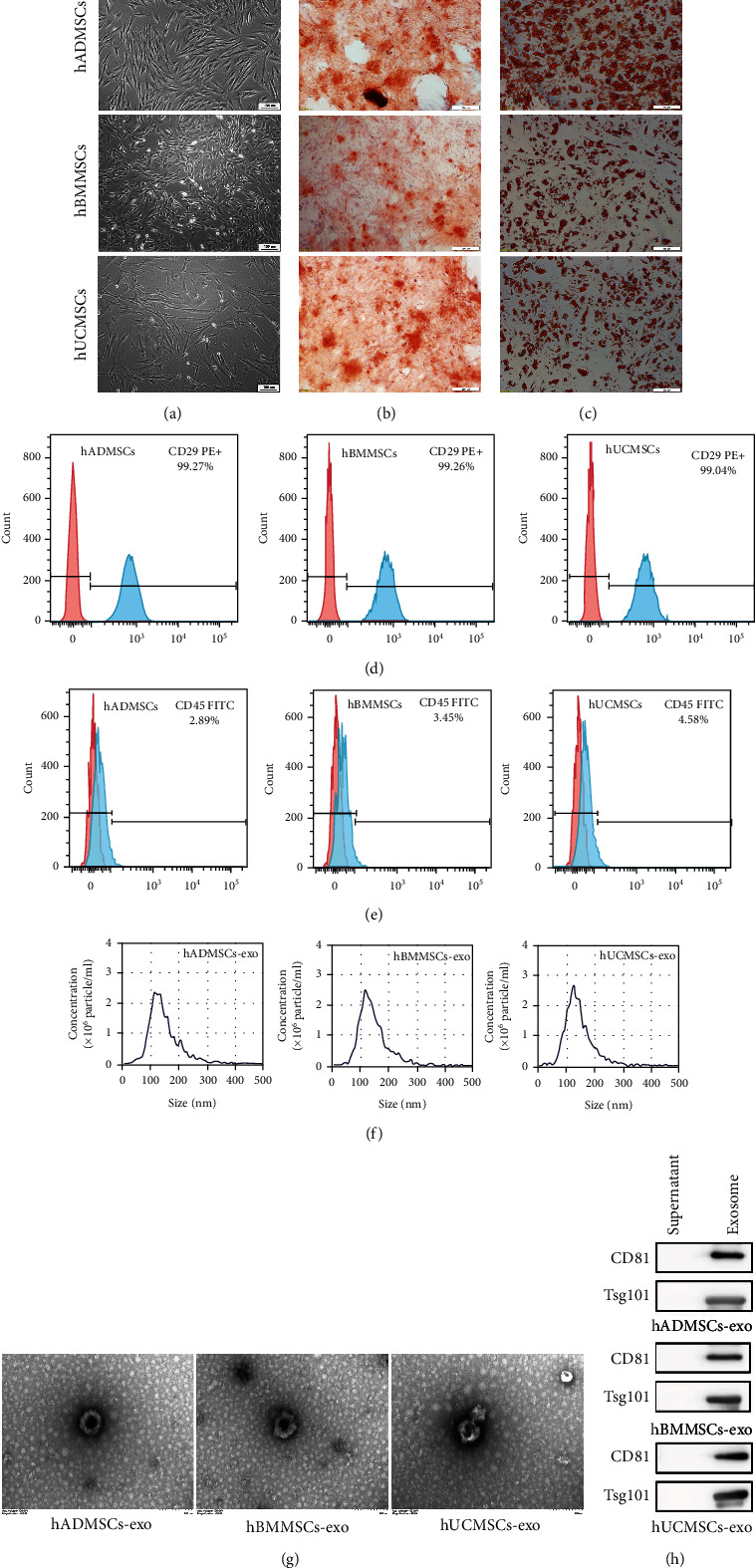
Characterization of human mesenchymal stem cells (hMSCs) and hMSC-derived exosomes (hMSC-exo). (a) Morphological observation of hMSCs derived from adipose tissue (hADMSCs), bone marrow (hBMMSCs), or umbilical cord (hUCMSCs). hMSCs had a long spindle shape and were arranged in an organized fashion. Scale bar, 100 *μ*m. (b, c) The multidifferentiation potential of hMSCs *in vitro*. Alizarin red S staining was used to evaluate (b) osteogenic differentiation, while oil red O staining was used to evaluate (c) adipogenic differentiation capacity. Scale bar, 100 *μ*m. (d, e) Flow cytometric analysis of hBMSC surface markers. Note that all hMSCs were (d) positive for CD29 and (e) negative for CD45. The red shape represents the target antibody, and the blue shape represents the isotype control antibody. (f) Nanoparticle tracking assay showed the size distribution of exosomes. (g) hADMSC-exo, hBMMSC-exo, and hUCMSC-exo under the transmission electron microscope. Scale bar, 200 nm. (h) Western blot showing that exosomes derived from the three types of hMSCs were TSG101- and CD81-positive.

All three types of hMSCs exhibited typical stem cell morphology resembling spindle-shaped fibroblasts (Figure 1(a)). All hMSCs were further characterized by confirming their ability to undergo specific osteogenic or adipogenic differentiation, as examined by alizarin red staining (Figure 1(b)) or oil red O staining (Figure 1(c)), respectively. Results of flow cytometry showed that all three hMSC types were positive for the mesenchymal stem cell marker CD29 (Figure 1(d)), while negative for the hematopoietic marker CD45 (Figure 1(e)). These observations confirmed that the hMSCs used in the study had typical stem cell characteristics.

Nanoparticle-tracking analysis showed that the diameters of all three types of hMSC-derived exosomes were in the range of 40-150 nm, with the highest concentration around 120 nm (Figure 1(f)). The three types of hMSC-derived exosomes exhibited a typical cup-shaped vesicle morphology under the transmission electron microscope, and no shape differences were noted among exosomes from the three tissue sources (Figure 1(g)). Western blot analysis confirmed that our extracted hMSC-derived exosomes expressed exosome markers such as CD81 and TSG101 (Figure 1(h)).

### 3.2. hMSCs Inhibited LPS-Induced Glycolysis in Macrophages and Their Production of Proinflammatory Cytokines

Our previous research demonstrated that LPS stimulation increases macrophage glycolysis and promotes macrophage polarization toward the M1 phenotype, thereby enhancing their release of proinflammatory cytokines and increasing inflammatory responses, while coculture with MSCs from mouse bone marrow effectively attenuates this process [9]. In the present study, we further evaluated the protective effect of hMSCs from different tissue sources on LPS-stimulated RAW264.7 cells.

**Figure 2:**
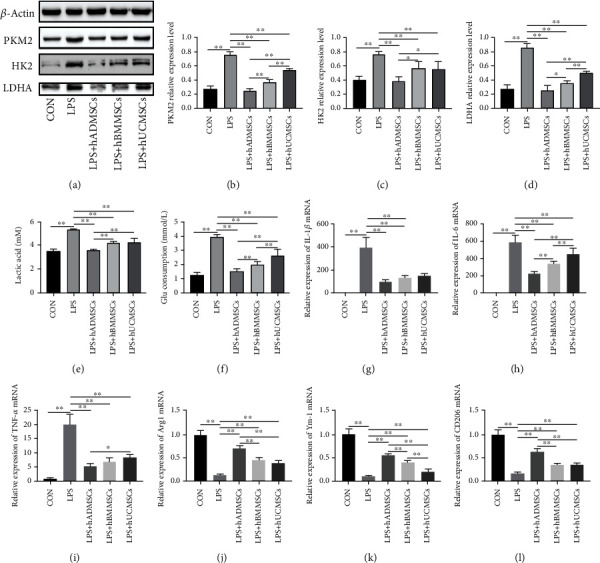
Human mesenchymal stem cells (hMSCs) inhibited lipopolysaccharide- (LPS-) induced glycolysis and proinflammatory cytokine production in macrophages. (a) Western blot experiment showed that hMSCs derived from adipose tissue (hADMSCs), bone marrow (hBMMSCs), and umbilical cord (hUCMSCs) inhibited key enzymes involved in glycolysis, including (b) PKM2, (c) HK2, and (d) LDHA. Moreover, the end products of glycolysis decreased, such as (e) lactic acid, as did (f) the consumption of glucose. hADMSCs, hBMMSCs, and hUCMSCs significantly reduced the mRNA expression of (g) interleukin- (IL-) 1*β*, (h) IL-6, and (i) tumor necrosis factor- (TNF-) *α* and increased the mRNA levels of (j) Arg1, (k) Ym-1, and (l) CD206 in LPS-treated RAW264.7 cells. All mRNA levels were normalized to the level of *β*-actin mRNA. Data are expressed as mean ± standard deviation (*n* = 6 in each group). *p* values were calculated using one-way ANOVA. ^∗^*p* < 0.05; ^∗∗^*p* < 0.01.

The results of western blot analyses showed that the protein levels of three essential enzymes in glycolysis, such as PKM2, HK2, and LDHA, increased after macrophage stimulation with 1 *μ*g/mL LPS for 24 h. Conversely, coculture with hMSCs from the three tissue sources significantly inhibited the expression of these three enzymes. hMSCs from adipose tissue had a more pronounced inhibitory effect than those from bone marrow or umbilical cord (Figures 2(a)–2(d)). In addition, after coculture with hMSCs, lactic acid production and glucose consumption in LPS-treated RAW264.7 cells decreased (Figures 2(e) and 2(f)).

Subsequently, we measured the mRNA expression levels of M1 polarization markers (IL-1*β*, IL-6, and TNF-*α*) and M2 polarization markers (Arg1, Ym-1, and CD206) by RT-qPCR. As shown in Figures 2(g)–2(l), LPS stimulation increased the mRNA expression of IL-1*β*, IL-6, and TNF-*α* in RAW264.7 cells compared with the control group, while coculture with hMSCs suppressed IL-1*β*, IL-6, and TNF-*α* mRNA expression. We also found that the expression of Arg1, Ym-1, and CD206 in RAW264.7 cells was significantly downregulated after LPS stimulation, while coculture with hMSCs increased their expression. Similarly, hMSCs from adipose tissue exhibited the most significant inhibitory effect on the expression of M2 polarization markers in LPS-treated cells. These results suggest that coculture with hMSCs reduces the production of inflammatory cytokines and inhibits the inflammatory response by downregulating the glycolysis levels in RAW264.7 cells, thus promoting their polarization toward M2 macrophages.

### 3.3. Exosomes from hMSCs Suppressed Glycolysis and Proinflammatory Cytokine Production in LPS-Treated Macrophages

**Figure 3:**
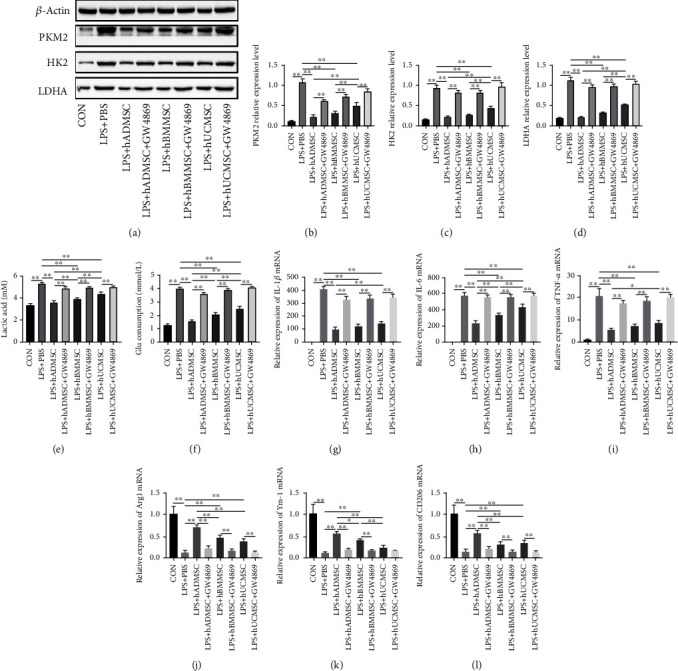
Human mesenchymal stem cells (hMSCs) suppressed glycolysis and production of proinflammatory cytokines in lipopolysaccharide- (LPS-) treated macrophages by secreting exosomes. (a) Western blot experiments showed that exosomes from hMSCs derived from adipose tissue (AD), bone marrow (hBMMSCs), and umbilical cord (hUCMSCs) inhibited key enzymes involved in glycolysis, including (b) PKM2, (c) HK2, and (d) LDHA. Moreover, the end products of glycolysis decreased, such as (e) lactic acid, as did (f) the consumption of glucose. The exosome inhibitor GW4869 eliminated the inhibitory effect. hADMSCs, hBMMSCs, and hUCMSCs significantly reduced the mRNA expression of (g) IL-1*β*, (h) IL-6, and (i) TNF-*α* and increased the mRNA levels of (j) Arg1, (k) Ym-1, and (l) CD206 in LPS-treated RAW264.7 cells. These effects were also reversed by the exosome inhibitor GW4869. All mRNA levels were normalized to the level of *β*-actin mRNA. Data are expressed as mean ± standard deviation (*n* = 6 in each group). *p* values were calculated by one-way ANOVA. ^∗^*p* < 0.05; ^∗∗^*p* < 0.01.

To investigate whether hMSCs exert their protective effect by secreting exosomes, we treated hMSCs with GW4869, an agent that inhibits secretion of exosomes. We found that pretreatment with GW4869 for 24 h nearly eliminated the inhibitory effect of hMSCs on the expression of key enzymes in glycolysis (PKM2, HK2, and LDHA) in LPS-treated RAW264.7 cells (Figures 3(a)–3(d)). Consistently, GW4869 also nearly eliminated the inhibitory effect of hMSCs on lactic acid production and glucose consumption in LPS-treated RAW264.7 cells (Figures 3(e) and 3(f)). The results of RT-qPCR showed that GW4869 pretreatment effectively inhibited the regulatory effect of hMSCs on the expression of M1 and M2 markers in LPS-treated RAW264.7 cells (Figures 3(g)–3(l)). The above results suggest that exosomes may mediate the ability of hMSCs to downregulate glycolysis and proinflammatory cytokine expression in LPS-treated macrophages.

### 3.4. hMSC-Derived Exosomes Suppressed Glycolysis and Proinflammatory Cytokine Production in LPS-Treated Macrophages

**Figure 4:**
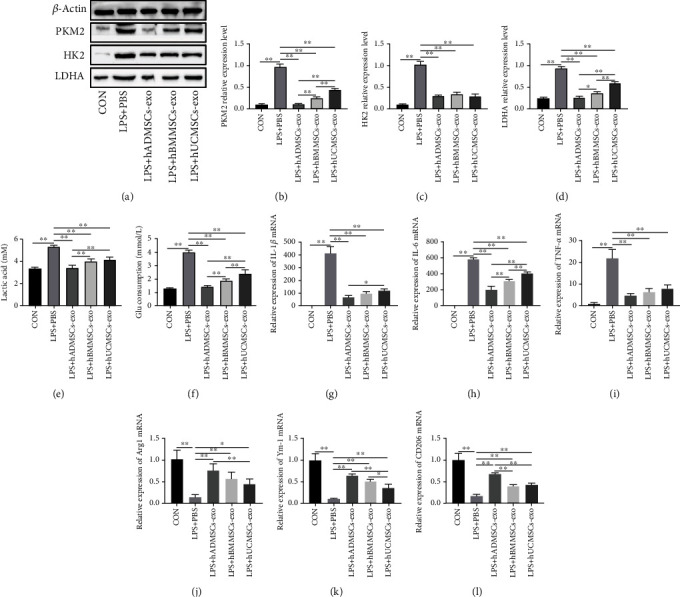
Human mesenchymal stem cell- (hMSC-) derived exosomes suppressed glycolysis and production of proinflammatory cytokines in lipopolysaccharide- (LPS-) treated macrophages. (a) Western blot experiments showed that exosomes from hMSCs derived from adipose tissue (hADMSCs), bone marrow (hBMMSCs), and umbilical cord (hUCMSCs) inhibited key involved in the glycolysis, including (b) PKM2, (c) HK2, and (d) LDHA. Moreover, the end products of glycolysis, such as (e) lactic acid, as well as (f) the consumption of glucose, decreased. hADMSC-derived exosomes, hBMMSC-derived exosomes, and hUCMSC-derived exosomes significantly reduced the mRNA expression of (g) IL-1*β*, (h) IL-6, and (i) TNF-*α* and increased the mRNA levels of (j) Arg1, (k) Ym-1, and (l) CD206 in LPS-treated RAW264.7 cells. All mRNA levels were normalized to the level of *β*-actin mRNA. The data are expressed as the mean ± standard deviation. *n* = 6 in each group. *p* values were calculated by one-way ANOVA. ^∗^*p* < 0.05; ^∗∗^*p* < 0.01.

To further clarify the role of hMSC-derived exosomes in inhibiting cellular glycolysis and proinflammatory cytokine production in LPS-treated macrophages, we extracted exosomes from the three types of hMSCs. We found that, consistent with the effect of coculture with hMSCs, coculture of LPS-stimulated RAW264.7 cells with exosomes inhibited the expression of PKM2, HK2, and LDHA. In particular, the inhibitory effect of exosomes derived from hMSCs from adipose tissue was greater than the effect of the other two types of exosomes (Figures 4(a)–4(d)). Similarly, hMSC-derived exosomes strongly reduced lactic acid production and glucose consumption in LPS-treated RAW264.7 cells (Figures 4(e) and 4(f)).

Next, we measured the expression of inflammatory cytokines at the mRNA level: all three types of hMSC-derived exosomes potently downregulated the expression of IL-1*β*, IL-6, and TNF-*α* in LPS-treated RAW264.7 cells, while they upregulated Arg1, Ym-1, and CD206 expression. Among the three types of hMSC-derived exosomes, those derived from adipose tissue hMSCs had the greatest effect on the expression of M1 and M2 markers in LPS-treated macrophages (Figures 4(g)–4(l)). These results confirmed that hMSC-derived exosomes exhibit similar beneficial effects as hMSCs on inhibiting glycolysis in LPS-treated RAW264.7 cells, promoting their polarization toward the M2 phenotype. This in turn decreases the production of proinflammatory cytokines and alleviates the inflammatory response.

### 3.5. hMSCs Alleviated Sepsis-Induced ALI and Systemic Inflammation and Improved Survival in Mice

**Figure 5:**
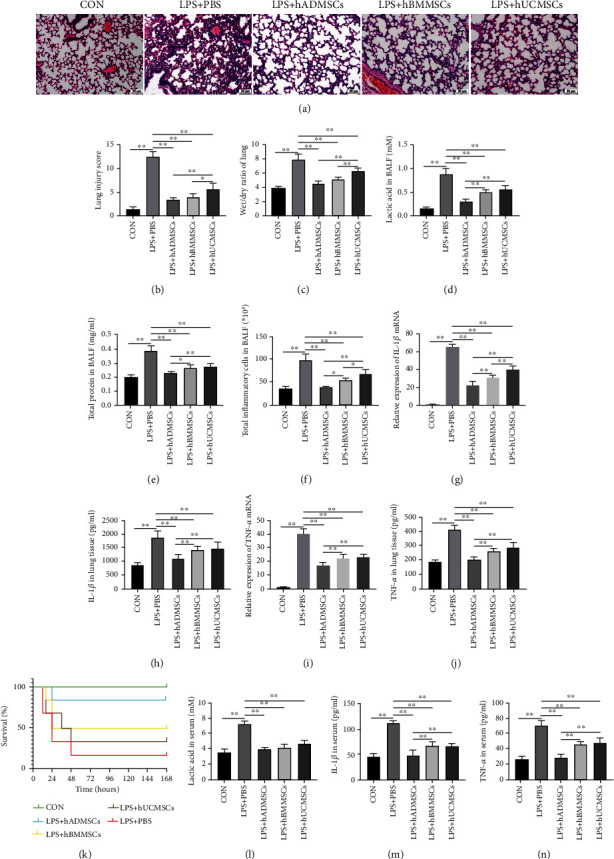
Human mesenchymal stem cells (hMSCs) alleviated sepsis-induced ALI and systemic inflammation and improved survival in mice. (a) Hematoxylin and eosin staining of lung tissue sections from the different experimental groups. (b) Lung injury score analysis. (c) Wet-to-dry ratio of lung tissues. (d) Lactic acid content in bronchoalveolar lavage fluid (BALF). (e) Protein concentration in BALF. (f) Inflammatory cell counts in BALF. mRNA expression of (g) interleukin- (IL-) 1*β* and (i) tumor necrosis factor- (TNF-) *α* in lung tissue. Levels of (h) IL-1*β* and (j) TNF-*α* in lung tissue measured by ELISA. (k) Survival of mice (*n* = 12 mice in each group); *p* < 0.001 among the curves as determined using the log-rank (Mantel-Cox) test. Levels of (l) lactic acid, (m) IL-1*β*, and (n) TNF-*α* in serum, as measured by ELISA. Data in (b–j) and (l–n) are expressed as mean ± standard deviation (*n* = 6 in each group); *p* values were calculated using one-way ANOVA. ^∗^*p* < 0.05; ^∗∗^*p* < 0.01.

To evaluate the therapeutic activity of hMSC-derived exosomes in sepsis-induced ALI, an *in vivo* sepsis model was established in mice by intraperitoneal injection of LPS (10 mg/kg). One hour after injection, 1 × 10^6^ hMSCs from one of the three tissue sources were injected via the tail vein, and lung injury and systemic inflammation were analyzed 24 h later. We found that, after LPS injection, the lung tissues of mice exhibited various features of acute injury, including incomplete alveolar wall, interstitial edema, and inflammatory cell infiltration, and all these signs were significantly alleviated after hMSC injection (Figure 5(a)). Consistently, lung injury scores and wet-to-dry ratios of lung tissues also decreased substantially after hMSC injection. The effects of hMSCs derived from adipose tissue and bone marrow were more significant than those of hMSCs derived from the umbilical cord (Figures 5(b) and 5(c)).

The results of BALF analysis showed that tail vein injection of 1 × 10^6^ hMSCs resulted in a significant decrease in lactic acid content, protein content, and total inflammatory cell count, with hMSCs from adipose tissue exerting the most prominent effects (Figures 5(d)–5(f)).

We also examined the expression and production of proinflammatory cytokines in lung tissues by RT-qPCR and ELISA and found that IL-1*β* and TNF-*α* were upregulated in lung tissues of LPS-injected mice. All three types of hMSCs downregulated the expression and production of IL-1*β* and TNF-*α* (Figures 5(g)–5(j)). In addition to improving intrapulmonary conditions in sepsis-induced ALI mice, hMSC injection also improved survival, lactic acid content, and systemic inflammatory condition of the animals (Figures 5(k)–5(n)). Again, hMSCs from adipose tissue showed the greatest protective effect. These *in vivo* experiments further confirmed that hMSCs attenuated sepsis-induced ALI in mice, reducing systemic inflammation and improving survival.

### 3.6. hMSC-Derived Exosomes Alleviated Sepsis-Induced ALI and Systemic Inflammation and Improved Survival in Mice

**Figure 6:**
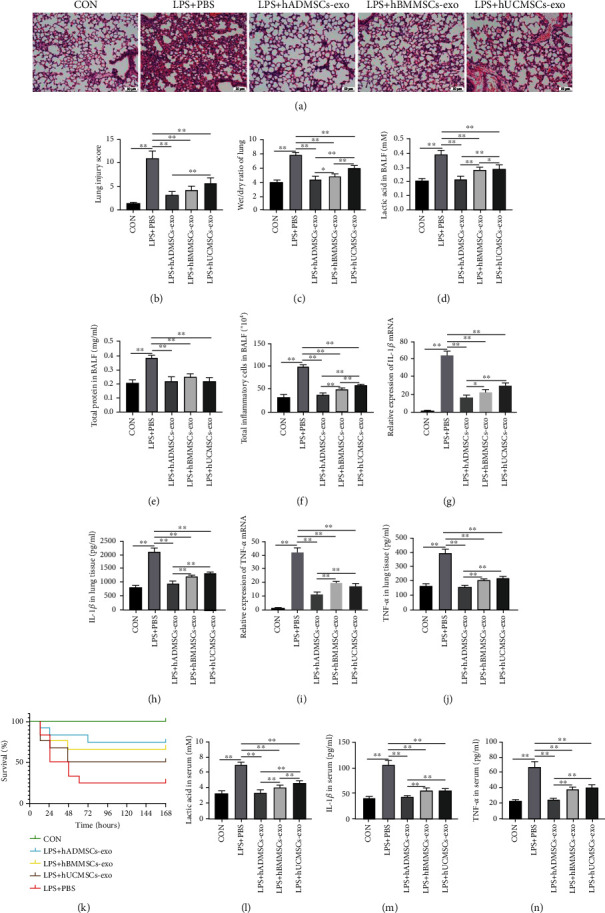
Human mesenchymal stem cell- (hMSC-) derived exosomes attenuated sepsis-induced acute lung injury and systemic inflammation and improved survival in mice. (a) Hematoxylin and eosin staining of lung tissue sections. (b) Lung injury score analysis. (c) Wet-to-dry ratio of lung tissues. (d) Lactic acid content in bronchoalveolar lavage fluid (BALF). (e) Protein concentration in BALF. (f) Inflammatory cell counts in BALF. mRNA expression of (g) interleukin- (IL-) 1*β* and (i) tumor necrosis factor- (TNF-) *α* in lung tissue. Levels of (h) IL-1*β* and (j) TNF-*α* in lung tissue, as measured by ELISA. (k) Survival rate of mice (*n* = 12 mice in each group); *p* = 0.0033 among the curves as determined using the log-rank (Mantel-Cox) test. Levels of (l) lactic acid, (m) IL-1*β*, and (n) TNF-*α* in mouse serum, as measured by ELISA. All data in (b–j) and (l–n) are expressed as mean ± standard deviation (*n* = 6 in each group); *p* values were calculated using one-way ANOVA. ^∗^*p* < 0.05; ^∗∗^*p* < 0.01.

Administering each of the three types of hMSC-derived exosomes to sepsis-induced ALI mice significantly alleviated alveolar wall incompleteness, interstitial lung edema, and inflammatory cell infiltration (Figure 6(a)), thereby reducing lung injury scores and lung wet-to-dry ratio (Figures 6(b) and 6(c)). BALF analysis revealed that hMSC-derived exosomes reduced lactic acid content, protein content, and total inflammatory cell count. Similar to the experiments above, exosomes from hMSCs derived from adipose tissue achieved the greatest reduction in lactic acid content and inflammatory cell infiltration in BALF (Figures 6(d)–6(f)).

All three types of hMSC-derived exosomes reduced the expression and production of IL-1*β* and TNF-*α* in sepsis-induced ALI mice, in particular exosomes from adipose tissue hMSCs (Figures 6(g)–6(j)). Administering hMSC-derived exosomes to sepsis-induced ALI mice improved their survival (Figure 6(k)). Serum lactic acid, IL-1*β*, and TNF-*α* decreased after the administration of hMSC-derived exosomes, particularly those derived from the adipose tissue (Figures 6(l)–6(n)). Collectively, the above results confirm that hMSC-derived exosomes can protect lung tissue, alleviate systemic inflammation, and improve survival in sepsis-induced ALI mice.

## 4. Discussion

Previous studies demonstrated that hMSCs and their derived exosomes protect against sepsis-induced ALI, but the exact type of hMSCs remained unclear. In the present work, we compared the lung therapeutic effects of hMSCs from adipose tissue, bone marrow, and umbilical cord as well as their secreted exosomes on macrophage polarization and sepsis-induced ALI *in vitro* and *in vivo*. We found that coculture of macrophages with the three types of hMSCs or their derived exosomes inhibited glycolysis, promoted polarization toward the M2 phenotype, and reduced the release of proinflammatory cytokines. In mice, the three types of hMSCs or their derived exosomes significantly alleviated the sepsis-induced inflammatory condition in lung tissues, reduced the systemic inflammatory response, and improved survival.

We found that hMSCs from the three tissue sources regulated macrophage polarization and suppressed inflammatory responses. Previous work demonstrated that MSCs exhibit potent organ protective effects, for example, on the heart [26], liver [27], kidney [28], retina [29], and brain [30]. Further studies also showed that the powerful protective effect of MSCs on various organs is largely dependent on the intercellular communication between MSCs and macrophages [31]. The polarization status of macrophages is directly related to their role in the inflammatory response and may involve JAK/STAT [32], PI3K/Akt [33], Notch [34], and JNK signaling pathways [35].

We also showed that hMSCs changed the metabolic state of macrophages by inhibiting their glycolysis, thus regulating their polarization state and function. Intracellular glycolytic metabolism provides metabolic intermediates for biosynthetic pathways such as ribose, amino acids, and fatty acids. In M1 macrophages, an efficient glycolysis process exists [36], which promotes the production of cellular inflammatory cytokines during the inflammatory response and maintains the sterilizing activity of M1 macrophages [37]. In M2 macrophages, in contrast, glucose metabolism occurs mainly through aerobic oxidation, and glycolysis is largely absent [38]. Recent studies suggest that when glycolysis is inhibited in macrophages, polarization to the M2 phenotype occurs, leading to anti-inflammatory and organ-protective effects [39, 40]. Consistent with these findings, our previous studies also confirmed that MSCs can alleviate inflammation by inhibiting macrophage glycolysis, thereby regulating macrophage polarization [9, 41, 42].

Recent studies have shown that hMSC-derived exosomes can play a critical therapeutic role in inflammatory diseases by regulating apoptosis and affecting cell proliferation [43–46]. In this study, we compared the protective effects of three different tissue-derived hMSCs and their released exosomes on sepsis-induced ALI, and our results demonstrated that all three kinds of exosomes could play a therapeutic role in regulating macrophage polarization and attenuating lung pathological injury. Exosomes derived from hMSCs from adipose tissue exhibited particularly strong effects in promoting macrophage M2 polarization, inhibiting proinflammatory cytokine production and secretion, attenuating lung histopathological changes, and improving survival of sepsis-induced ALI mice. Therefore, exosomes derived from hMSCs from adipose tissue deserve more attention in future studies, which should examine how they exert their therapeutic effects.

The use of MSC-derived exosomes has shown excellent promise for the treatment of sepsis-induced ALI or ARDS. The various components of MSC-derived exosomes can exert anti-inflammatory effects and antiapoptotic effects and promote cell regeneration [47–50]. For example, such exosomes may exert protective effects against sepsis-induced ALI or ARDS by transferring nucleic acids including mRNAs, long noncoding RNAs, and microRNAs [21] that modulate the MAPK signaling pathway to affect the levels of proinflammatory cytokines in the lung tissue [23] and by transferring other biological components such as mitochondrial or intracellular proteins [51]. Our study suggests that hMSCs from the three tissue sources, in particular those derived from the adipose tissue, and their secreted exosomes can exert pulmonary protective effects by inhibiting glycolysis of alveolar macrophages, thereby promoting macrophage M2 polarization. In future studies, we will focus on adipose tissue-hMSC-derived exosomes and analyze in more detail how they counteract sepsis-induced ALI.

## 5. Conclusions

In summary, our present findings reveal that hMSC-derived exosomes can effectively downregulate sepsis-induced glycolysis and inflammation in macrophages, ameliorate lung pathology, and improve survival of mice with sepsis. Notably, the protective effects of hMSCs and their exosomes from adipose tissue were more obvious than those derived from bone marrow and umbilical cord.

## Figures and Tables

**Table 1:** Primer sequences used for reverse transcription quantitative real-time PCR assays.

Gene	Primer	Sequence
*β*-Actin	Forward	AGTGTGACGTTGACATCCGT
	Reverse	GCAGCTCAGTAACAGTCCGC
IL-1*β*	Forward	GCAACTGTTCCTGAACTCAACT
	Reverse	ATCTTTTGG GGTCCGTCAACT
IL-6	Forward	TAGTCCTTCCTACCCCAATTTCC
	Reverse	TTGGTCCTTAGCCACTCCTTC
TNF-*α*	Forward	AAGCCTGTAGCCCACGTCGTA
	Reverse	GGCACCACTAGTTGGTTGTCTTTG
Arg1	Forward	CTCCAAGCCAAAGTCCTTAGAG
	Reverse	GGAGCTGTCATTAGGGACATCA
Ym-1	Forward	CCAAGTGCAGCATGTGTCAG
	Reverse	CCTCTACGTTCCCCAAGTCG
CD206	Forward	CTCTGTTCAGCTATTGGACGC
	Reverse	CGGAATTTCTGGGATTCAGCTTC

## Data Availability

The datasets generated for this study are available on request to the corresponding author.

## Abbreviations

hMSCs: Human mesenchymal stem cells
ARDS: Acute respiratory distress syndrome
LPS: Lipopolysaccharide
ALI: Acute lung injury
IL: Interleukin
TNF: Tumor necrosis factor
MSCs: Mesenchymal stem cells
PBS: Phosphate-buffered saline
BALF: Bronchoalveolar lavage fluid
RT-qPCR: Reverse transcription quantitative real-time PCR
PKM2: Pyruvate kinase isoform M2
HK2: Hexokinase 2
LDHA: Lactic acid dehydrogenase A
ELISA: Enzyme-linked immunosorbent assay.
